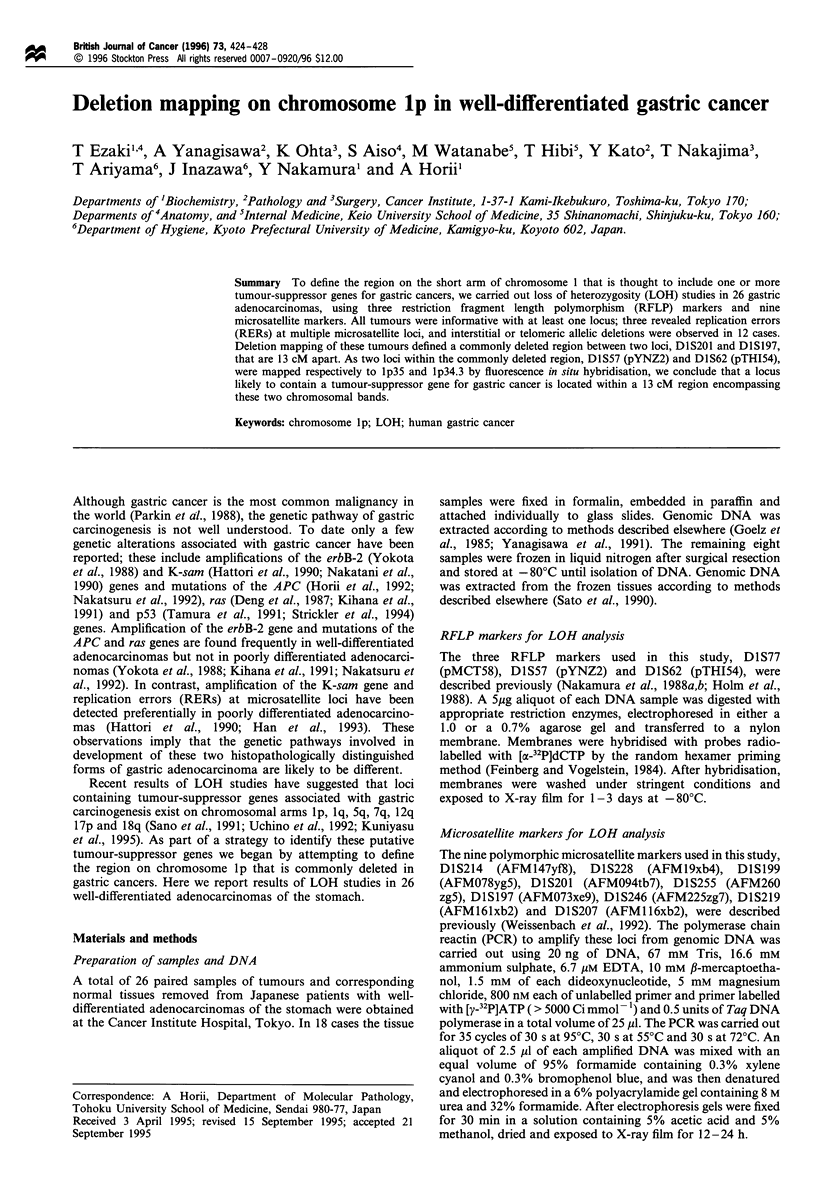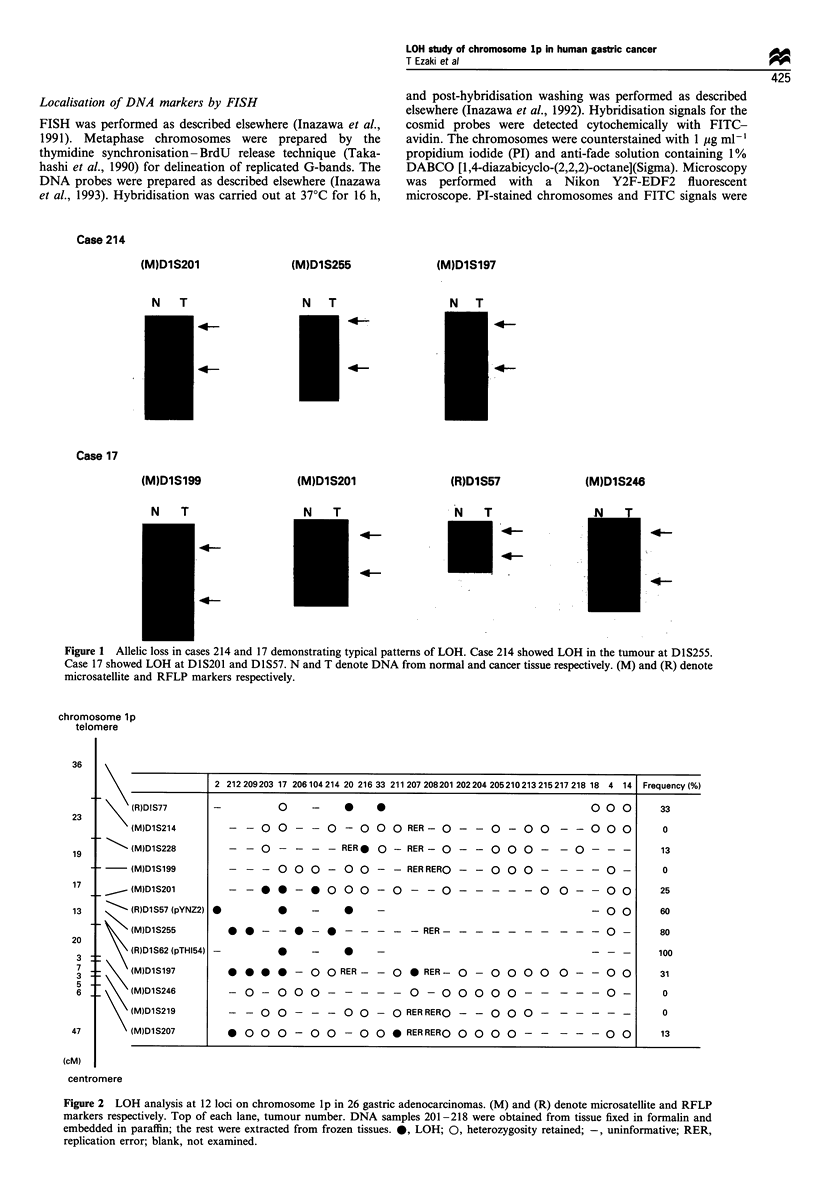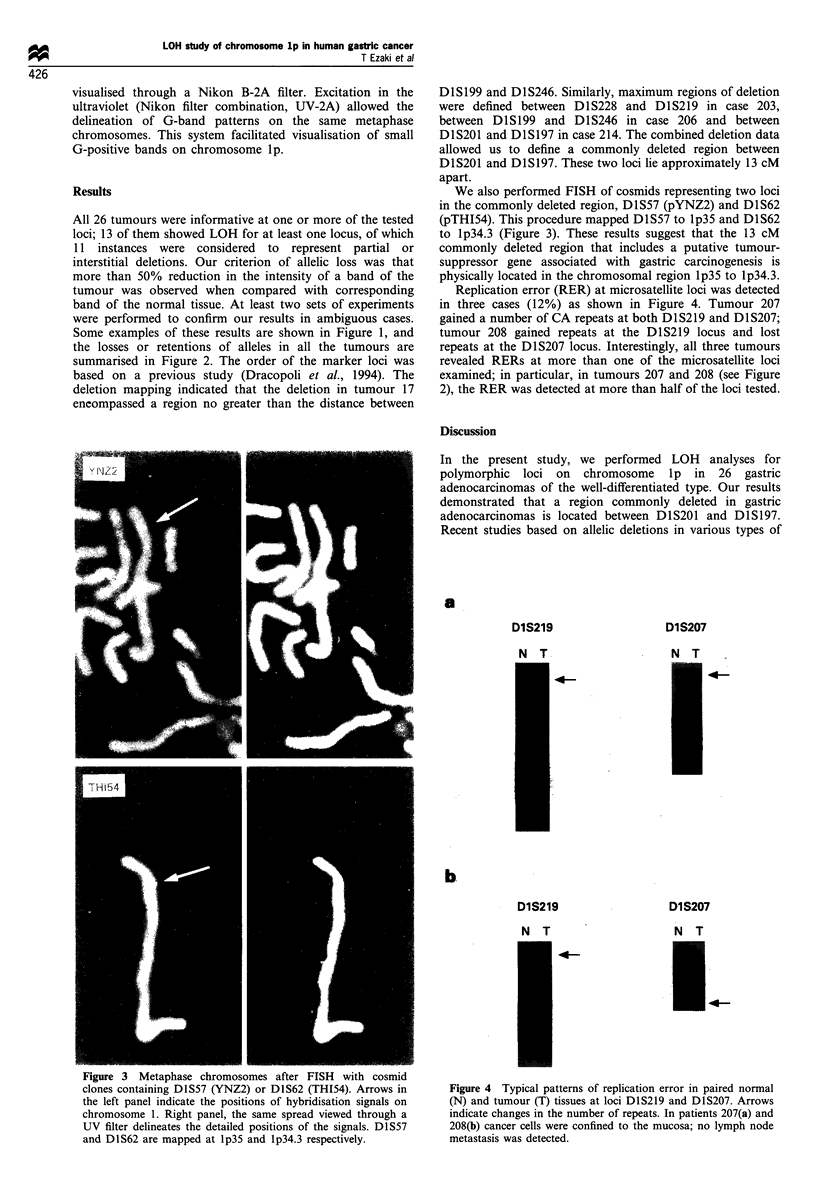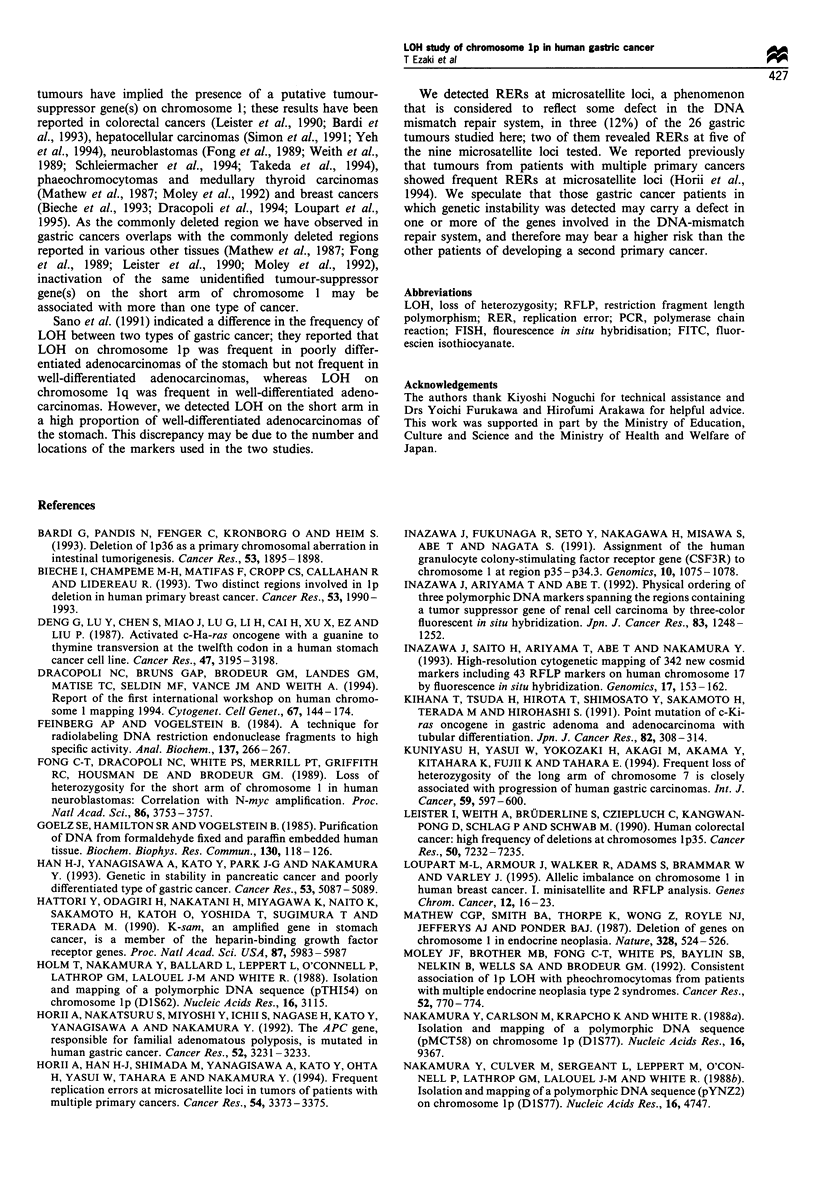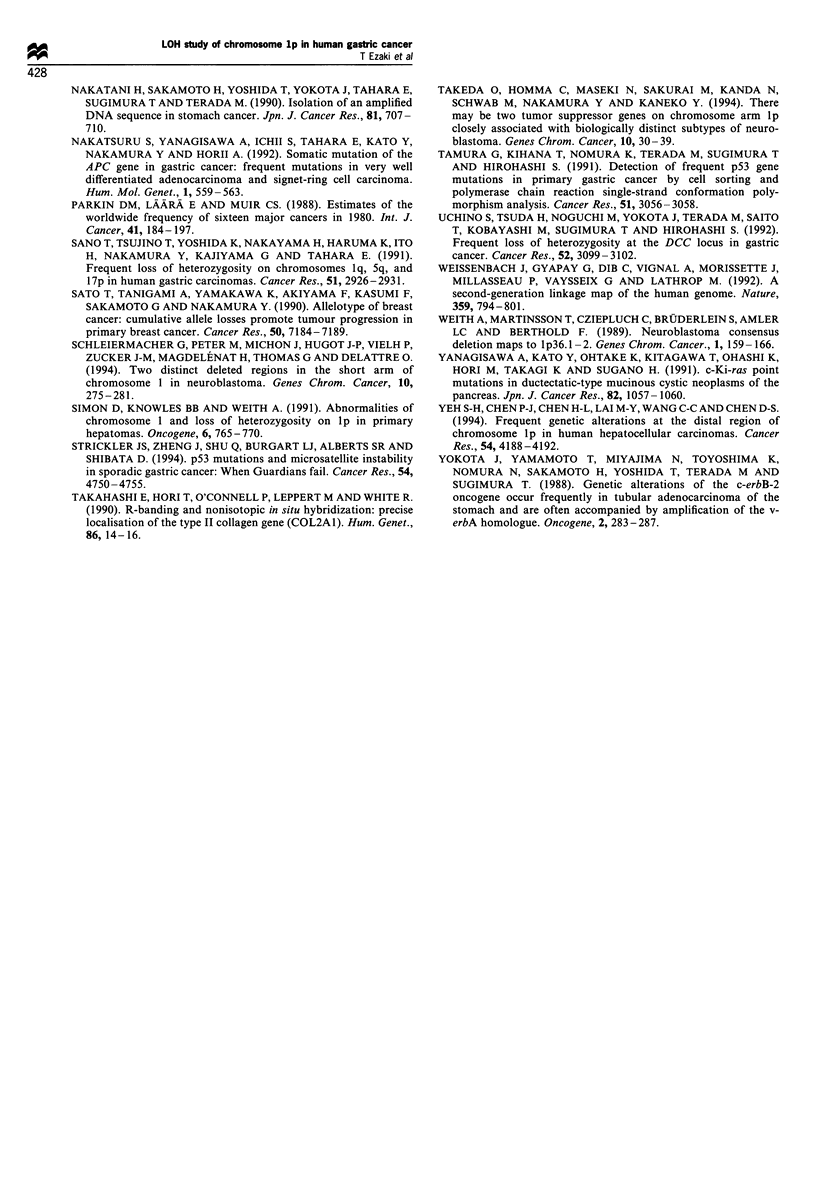# Deletion mapping on chromosome 1p in well-differentiated gastric cancer.

**DOI:** 10.1038/bjc.1996.76

**Published:** 1996-02

**Authors:** T. Ezaki, A. Yanagisawa, K. Ohta, S. Aiso, M. Watanabe, T. Hibi, Y. Kato, T. Nakajima, T. Ariyama, J. Inazawa, Y. Nakamura, A. Horii

**Affiliations:** Department of Biochemistry, Cancer Institute, Tokyo, Japan.

## Abstract

**Images:**


British Journal of Cancer (1996) 73, 424-428
© 1996 Stockton Press All rights reserved 0007-0920/96 $12.00

### Deletion mapping on chromosome 1p in well-differentiated gastric cancer

T Ezaki[1,4], A Yanagisawa[2], K Ohta[3], S Aiso[4], M Watanabe[5], T Hibi[5], Y Kato[2], T Nakajima[3], T Ariyama[6], J Inazawa[6], Y Nakamura[1] and A Horii[1]

*Departments of [1]Biochemistry, [2]Pathology and [3]Surgery, Cancer Institute, 1-37-1 Kami-Ikebukuro, Toshima-ku, Tokyo 170; Deparments of [4]Anatomy, and [5]Internal Medicine, Keio University School of Medicine, 35 Shinanomachi, Shinjuku-ku, Tokyo 160; [6]Department of Hygiene, Kyoto Prefectural University of Medicine, Kamigyo-ku, Koyoto 602, Japan.*

**Summary** To define the region on the short arm of chromosome 1 that is thought to include one or more tumour-suppressor genes for gastric cancers, we carried out loss of heterozygosity (LOH) studies in 26 gastric adenocarcinomas, using three restriction fragment length polymorphism (RFLP) markers and nine microsatellite markers. All tumours were informative with at least one locus; three revealed replication errors (RERs) at multiple microsatellite loci, and interstitial or telomeric allelic deletions were observed in 12 cases. Deletion mapping of these tumours defined a commonly deleted region between two loci, D1S201 and D1S197, that are 13 cM apart. As two loci within the commonly deleted region, D1S57 (pYNZ2) and D1S62 (pTHI54), were mapped respectively to 1p35 and 1p34.3 by fluorescence *in situ* hybridisation, we conclude that a locus likely to contain a tumour-suppressor gene for gastric cancer is located within a 13 cM region encompassing these two chromosomal bands.

**Keywords:** chromosome 1p; LOH; human gastric cancer

Although gastric cancer is the most common malignancy in the world (Parkin *et al.*, 1988), the genetic pathway of gastric carcinogenesis is not well understood. To date only a few genetic alterations associated with gastric cancer have been reported; these include amplifications of the *erb*B-2 (Yokota *et al.*, 1988) and K-*sam* (Hattori *et al.*, 1990; Nakatani *et al.*, 1990) genes and mutations of the *APC* (Horii *et al.*, 1992; Nakatsuru *et al.*, 1992), *ras* (Deng *et al.*, 1987; Kihana *et al.*, 1991) and p53 (Tamura *et al.*, 1991; Strickler *et al.*, 1994) genes. Amplification of the *erb*B-2 gene and mutations of the *APC* and *ras* genes are found frequently in well-differentiated adenocarcinomas but not in poorly differentiated adenocarcinomas (Yokota *et al.*, 1988; Kihana *et al.*, 1991; Nakatsuru *et al.*, 1992). In contrast, amplification of the K-*sam* gene and replication errors (RERs) at microsatellite loci have been detected preferentially in poorly differentiated adenocarcinomas (Hattori *et al.*, 1990; Han *et al.*, 1993). These observations imply that the genetic pathways involved in development of these two histopathologically distinguished forms of gastric adenocarcinoma are likely to be different.

Recent results of LOH studies have suggested that loci containing tumour-suppressor genes associated with gastric carcinogenesis exist on chromosomal arms 1p, 1q, 5q, 7q, 12q 17p and 18q (Sano *et al.*, 1991; Uchino *et al.*, 1992; Kuniyasu *et al.*, 1995). As part of a strategy to identify these putative tumour-suppressor genes we began by attempting to define the region on chromosome 1p that is commonly deleted in gastric cancers. Here we report results of LOH studies in 26 well-differentiated adenocarcinomas of the stomach.

### Materials and methods

### *Preparation of samples and DNA*

A total of 26 paired samples of tumours and corresponding normal tissues removed from Japanese patients with well-differentiated adenocarcinomas of the stomach were obtained at the Cancer Institute Hospital, Tokyo. In 18 cases the tissue samples were fixed in formalin, embedded in paraffin and attached individually to glass slides. Genomic DNA was extracted according to methods described elsewhere (Goelz *et al.*, 1985; Yanagisawa *et al.*, 1991). The remaining eight samples were frozen in liquid nitrogen after surgical resection and stored at −80°C until isolation of DNA. Genomic DNA was extracted from the frozen tissues according to methods described elsewhere (Sato *et al.*, 1990).

### *RFLP markers for LOH analysis*

The three RFLP markers used in this study, D1S77 (pMCT58), D1S57 (pYNZ2) and D1S62 (pTHI54), were described previously (Nakamura *et al.*, 1988*a,b*; Holm *et al.*, 1988). A 5μg aliquot of each DNA sample was digested with appropriate restriction enzymes, electrophoresed in either a 1.0 or a 0.7% agarose gel and transferred to a nylon membrane. Membranes were hybridised with probes radiolabelled with [α-$^{32}$P]dCTP by the random hexamer priming method (Feinberg and Vogelstein, 1984). After hybridisation, membranes were washed under stringent conditions and exposed to X-ray film for 1−3 days at −80°C.

### *Microsatellite markers for LOH analysis*

The nine polymorphic microsatellite markers used in this study, D1S214 (AFM147yf8), D1S228 (AFM19xb4), D1S199 (AFM078yg5), D1S201 (AFM094tb7), D1S255 (AFM260 zg5), D1S197 (AFM073xe9), D1S246 (AFM225zg7), D1S219 (AFM161xb2) and D1S207 (AFM116xb2), were described previously (Weissenbach *et al.*, 1992). The polymerase chain reactin (PCR) to amplify these loci from genomic DNA was carried out using 20 ng of DNA, 67 mM Tris, 16.6 mM ammonium sulphate, 6.7 μM EDTA, 10 mM β-mercaptoethanol, 1.5 mM of each dideoxynucleotide, 5 mM magnesium chloride, 800 nM each of unlabelled primer and primer labelled with [γ-$^{32}$P]ATP (> 5000 Ci mmol$^{-1}$) and 0.5 units of *Taq* DNA polymerase in a total volume of 25 μl. The PCR was carried out for 35 cycles of 30 s at 95°C, 30 s at 55°C and 30 s at 72°C. An aliquot of 2.5 μl of each amplified DNA was mixed with an equal volume of 95% formamide containing 0.3% xylene cyanol and 0.3% bromophenol blue, and was then denatured and electrophoresed in a 6% polyacrylamide gel containing 8 M urea and 32% formamide. After electrophoresis gels were fixed for 30 min in a solution containing 5% acetic acid and 5% methanol, dried and exposed to X-ray film for 12−24 h.

Correspondence: A Horii, Department of Molecular Pathology, Tohoku University School of Medicine, Sendai 980-77, Japan
Received 3 April 1995; revised 15 September 1995; accepted 21 September 1995

*Localisation of DNA markers by FISH*

FISH was performed as described elsewhere (Inazawa *et al.*, 1991). Metaphase chromosomes were prepared by the thymidine synchronisation–BrdU release technique (Takahashi *et al.*, 1990) for delineation of replicated G-bands. The DNA probes were prepared as described elsewhere (Inazawa *et al.*, 1993). Hybridisation was carried out at 37°C for 16 h, and post-hybridisation washing was performed as described elsewhere (Inazawa *et al.*, 1992). Hybridisation signals for the cosmid probes were detected cytochemically with FITC–avidin. The chromosomes were counterstained with 1 $\mu g\ ml^{-1}$ propidium iodide (PI) and anti-fade solution containing 1% DABCO [1,4-diazabicyclo-(2,2,2)-octane](Sigma). Microscopy was performed with a Nikon Y2F-EDF2 fluorescent microscope. PI-stained chromosomes and FITC signals were

**Figure 1** Allelic loss in cases 214 and 17 demonstrating typical patterns of LOH. Case 214 showed LOH in the tumour at D1S255. Case 17 showed LOH at D1S201 and D1S57. N and T denote DNA from normal and cancer tissue respectively. (M) and (R) denote microsatellite and RFLP markers respectively.

| Marker | 2 | 212 | 209 | 203 | 17 | 206 | 104 | 214 | 20 | 216 | 33 | 211 | 207 | 208 | 201 | 202 | 204 | 205 | 210 | 213 | 215 | 217 | 218 | 18 | 4 | 14 | Frequency (%) |
|---|---|---|---|---|---|---|---|---|---|---|---|---|---|---|---|---|---|---|---|---|---|---|---|---|---|---|---|
| (R)D1S77 | – | | | | ○ | | – | | ● | | ● | | | | | | | | | | | | | ○ | ○ | ○ | 33 |
| (M)D1S214 | | – | – | ○ | ○ | – | – | ○ | – | ○ | ○ | ○ | RER | – | ○ | – | – | ○ | – | ○ | ○ | – | – | ○ | ○ | ○ | 0 |
| (M)D1S228 | | – | – | ○ | – | – | – | – | RER | ● | ○ | – | RER | – | ○ | – | – | ○ | ○ | ○ | – | – | ○ | – | – | – | 13 |
| (M)D1S199 | | – | – | – | ○ | ○ | ○ | – | ○ | ○ | – | – | RER | RER | ○ | – | – | ○ | ○ | ○ | – | – | – | – | ○ | – | 0 |
| (M)D1S201 | | – | – | ● | ● | – | ● | ○ | ○ | ○ | – | ○ | – | – | ○ | – | – | – | – | – | ○ | ○ | – | – | ○ | ○ | 25 |
| (R)D1S57 (pYNZ2) | ● | | | | ● | | – | | ● | | – | | | | | | | | | | | | | – | ○ | ○ | 60 |
| (M)D1S255 | | ● | ● | – | – | ● | – | ● | – | – | – | – | – | RER | – | – | – | – | – | – | – | – | – | – | ○ | – | 80 |
| (R)D1S62 (pTHI54) | – | | | | ● | | – | | ● | | – | | | | | | | | | | | | | – | – | – | 100 |
| (M)D1S197 | | ● | ● | ● | ● | – | ○ | ○ | RER | – | – | ○ | ● | RER | – | ○ | – | ○ | ○ | ○ | ○ | ○ | – | – | ○ | ○ | 31 |
| (M)D1S246 | | – | ○ | – | ○ | ○ | ○ | – | – | – | – | – | ○ | – | ○ | ○ | ○ | ○ | ○ | – | – | – | – | – | ○ | – | 0 |
| (M)D1S219 | | – | – | ○ | ○ | – | – | – | ○ | ○ | – | ○ | RER | RER | ○ | – | – | ○ | ○ | ○ | – | – | – | – | – | – | 0 |
| (M)D1S207 | | ● | ○ | ○ | ○ | – | ○ | ○ | – | ○ | ○ | ● | RER | RER | ○ | ○ | ○ | ○ | ○ | – | – | – | – | – | ○ | ○ | 13 |

**Figure 2** LOH analysis at 12 loci on chromosome 1p in 26 gastric adenocarcinomas. (M) and (R) denote microsatellite and RFLP markers respectively. Top of each lane, tumour number. DNA samples 201–218 were obtained from tissue fixed in formalin and embedded in paraffin; the rest were extracted from frozen tissues. ●, LOH; ○, heterozygosity retained; –, uninformative; RER, replication error; blank, not examined.

visualised through a Nikon B-2A filter. Excitation in the ultraviolet (Nikon filter combination, UV-2A) allowed the delineation of G-band patterns on the same metaphase chromosomes. This system facilitated visualisation of small G-positive bands on chromosome 1p.

### Results

All 26 tumours were informative at one or more of the tested loci; 13 of them showed LOH for at least one locus, of which 11 instances were considered to represent partial or interstitial deletions. Our criterion of allelic loss was that more than 50% reduction in the intensity of a band of the tumour was observed when compared with corresponding band of the normal tissue. At least two sets of experiments were performed to confirm our results in ambiguous cases. Some examples of these results are shown in Figure 1, and the losses or retentions of alleles in all the tumours are summarised in Figure 2. The order of the marker loci was based on a previous study (Dracopoli *et al.*, 1994). The deletion mapping indicated that the deletion in tumour 17 encompassed a region no greater than the distance between D1S199 and D1S246. Similarly, maximum regions of deletion were defined between D1S228 and D1S219 in case 203, between D1S199 and D1S246 in case 206 and between D1S201 and D1S197 in case 214. The combined deletion data allowed us to define a commonly deleted region between D1S201 and D1S197. These two loci lie approximately 13 cM apart.

We also performed FISH of cosmids representing two loci in the commonly deleted region, D1S57 (pYNZ2) and D1S62 (pTHI54). This procedure mapped D1S57 to 1p35 and D1S62 to 1p34.3 (Figure 3). These results suggest that the 13 cM commonly deleted region that includes a putative tumour-suppressor gene associated with gastric carcinogenesis is physically located in the chromosomal region 1p35 to 1p34.3.

Replication error (RER) at microsatellite loci was detected in three cases (12%) as shown in Figure 4. Tumour 207 gained a number of CA repeats at both D1S219 and D1S207; tumour 208 gained repeats at the D1S219 locus and lost repeats at the D1S207 locus. Interestingly, all three tumours revealed RERs at more than one of the microsatellite loci examined; in particular, in tumours 207 and 208 (see Figure 2), the RER was detected at more than half of the loci tested.

**Figure 3** Metaphase chromosomes after FISH with cosmid clones containing D1S57 (YNZ2) or D1S62 (THI54). Arrows in the left panel indicate the positions of hybridisation signals on chromosome 1. Right panel, the same spread viewed through a UV filter delineates the detailed positions of the signals. D1S57 and D1S62 are mapped at 1p35 and 1p34.3 respectively.

### Discussion

In the present study, we performed LOH analyses for polymorphic loci on chromosome 1p in 26 gastric adenocarcinomas of the well-differentiated type. Our results demonstrated that a region commonly deleted in gastric adenocarcinomas is located between D1S201 and D1S197. Recent studies based on allelic deletions in various types of

**Figure 4** Typical patterns of replication error in paired normal (N) and tumour (T) tissues at loci D1S219 and D1S207. Arrows indicate changes in the number of repeats. In patients 207(**a**) and 208(**b**) cancer cells were confined to the mucosa; no lymph node metastasis was detected.

tumours have implied the presence of a putative tumour-suppressor gene(s) on chromosome 1; these results have been reported in colorectal cancers (Leister *et al.*, 1990; Bardi *et al.*, 1993), hepatocellular carcinomas (Simon *et al.*, 1991; Yeh *et al.*, 1994), neuroblastomas (Fong *et al.*, 1989; Weith *et al.*, 1989; Schleiermacher *et al.*, 1994; Takeda *et al.*, 1994), phaeochromocytomas and medullary thyroid carcinomas (Mathew *et al.*, 1987; Moley *et al.*, 1992) and breast cancers (Bieche *et al.*, 1993; Dracopoli *et al.*, 1994; Loupart *et al.*, 1995). As the commonly deleted region we have observed in gastric cancers overlaps with the commonly deleted regions reported in various other tissues (Mathew *et al.*, 1987; Fong *et al.*, 1989; Leister *et al.*, 1990; Moley *et al.*, 1992), inactivation of the same unidentified tumour-suppressor gene(s) on the short arm of chromosome 1 may be associated with more than one type of cancer.

Sano *et al.* (1991) indicated a difference in the frequency of LOH between two types of gastric cancer; they reported that LOH on chromosome 1p was frequent in poorly differentiated adenocarcinomas of the stomach but not frequent in well-differentiated adenocarcinomas, whereas LOH on chromosome 1q was frequent in well-differentiated adenocarcinomas. However, we detected LOH on the short arm in a high proportion of well-differentiated adenocarcinomas of the stomach. This discrepancy may be due to the number and locations of the markers used in the two studies.

We detected RERs at microsatellite loci, a phenomenon that is considered to reflect some defect in the DNA mismatch repair system, in three (12%) of the 26 gastric tumours studied here; two of them revealed RERs at five of the nine microsatellite loci tested. We reported previously that tumours from patients with multiple primary cancers showed frequent RERs at microsatellite loci (Horii *et al.*, 1994). We speculate that those gastric cancer patients in which genetic instability was detected may carry a defect in one or more of the genes involved in the DNA-mismatch repair system, and therefore may bear a higher risk than the other patients of developing a second primary cancer.

**Abbreviations**

LOH, loss of heterozygosity; RFLP, restriction fragment length polymorphism; RER, replication error; PCR, polymerase chain reaction; FISH, flourescence *in situ* hybridisation; FITC, fluorescien isothiocyanate.

**Acknowledgements**

The authors thank Kiyoshi Noguchi for technical assistance and Drs Yoichi Furukawa and Hirofumi Arakawa for helpful advice. This work was supported in part by the Ministry of Education, Culture and Science and the Ministry of Health and Welfare of Japan.

### References

BARDI G, PANDIS N, FENGER C, KRONBORG O AND HEIM S. (1993). Deletion of 1p36 as a primary chromosomal aberration in intestinal tumorigenesis. *Cancer Res.*, **53**, 1895–1898.

BIECHE I, CHAMPEME M-H, MATIFAS F, CROPP CS, CALLAHAN R AND LIDEREAU R. (1993). Two distinct regions involved in 1p deletion in human primary breast cancer. *Cancer Res.*, **53**, 1990–1993.

DENG G, LU Y, CHEN S, MIAO J, LU G, LI H, CAI H, XU X, EZ AND LIU P. (1987). Activated c-Ha-*ras* oncogene with a guanine to thymine transversion at the twelfth codon in a human stomach cancer cell line. *Cancer Res.*, **47**, 3195–3198.

DRACOPOLI NC, BRUNS GAP, BRODEUR GM, LANDES GM, MATISE TC, SELDIN MF, VANCE JM AND WEITH A. (1994). Report of the first international workshop on human chromosome 1 mapping 1994. *Cytogenet. Cell Genet.*, **67**, 144–174.

FEINBERG AP AND VOGELSTEIN B. (1984). A technique for radiolabeling DNA restriction endonuclease fragments to high specific activity. *Anal. Biochem.*, **137**, 266–267.

FONG C-T, DRACOPOLI NC, WHITE PS, MERRILL PT, GRIFFITH RC, HOUSMAN DE AND BRODEUR GM. (1989). Loss of heterozygosity for the short arm of chromosome 1 in human neuroblastomas: Correlation with N-*myc* amplification. *Proc. Natl Acad. Sci.*, **86**, 3753–3757.

GOELZ SE, HAMILTON SR AND VOGELSTEIN B. (1985). Purification of DNA from formaldehyde fixed and paraffin embedded human tissue. *Biochem. Biophys. Res. Commun.*, **130**, 118–126.

HAN H-J, YANAGISAWA A, KATO Y, PARK J-G AND NAKAMURA Y. (1993). Genetic in stability in pancreatic cancer and poorly differentiated type of gastric cancer. *Cancer Res.*, **53**, 5087–5089.

HATTORI Y, ODAGIRI H, NAKATANI H, MIYAGAWA K, NAITO K, SAKAMOTO H, KATOH O, YOSHIDA T, SUGIMURA T AND TERADA M. (1990). K-*sam*, an amplified gene in stomach cancer, is a member of the heparin-binding growth factor receptor genes. *Proc. Natl Acad. Sci. USA*, **87**, 5983–5987

HOLM T, NAKAMURA Y, BALLARD L, LEPPERT L, O'CONNELL P, LATHROP GM, LALOUEL J-M AND WHITE R. (1988). Isolation and mapping of a polymorphic DNA sequence (pTHI54) on chromosome 1p (D1S62). *Nucleic Acids Res.*, **16**, 3115.

HORII A, NAKATSURU S, MIYOSHI Y, ICHII S, NAGASE H, KATO Y, YANAGISAWA A AND NAKAMURA Y. (1992). The *APC* gene, responsible for familial adenomatous polyposis, is mutated in human gastric cancer. *Cancer Res.*, **52**, 3231–3233.

HORII A, HAN H-J, SHIMADA M, YANAGISAWA A, KATO Y, OHTA H, YASUI W, TAHARA E AND NAKAMURA Y. (1994). Frequent replication errors at microsatellite loci in tumors of patients with multiple primary cancers. *Cancer Res.*, **54**, 3373–3375.

INAZAWA J, FUKUNAGA R, SETO Y, NAKAGAWA H, MISAWA S, ABE T AND NAGATA S. (1991). Assignment of the human granulocyte colony-stimulating factor receptor gene (CSF3R) to chromosome 1 at region p35–p34.3. *Genomics*, **10**, 1075–1078.

INAZAWA J, ARIYAMA T AND ABE T. (1992). Physical ordering of three polymorphic DNA markers spanning the regions containing a tumor suppressor gene of renal cell carcinoma by three-color fluorescent *in situ* hybridization. *Jpn. J. Cancer Res.*, **83**, 1248–1252.

INAZAWA J, SAITO H, ARIYAMA T, ABE T AND NAKAMURA Y. (1993). High-resolution cytogenetic mapping of 342 new cosmid markers including 43 RFLP markers on human chromosome 17 by fluorescence *in situ* hybridization. *Genomics*, **17**, 153–162.

KIHANA T, TSUDA H, HIROTA T, SHIMOSATO Y, SAKAMOTO H, TERADA M AND HIROHASHI S. (1991). Point mutation of c-Ki-*ras* oncogene in gastric adenoma and adenocarcinoma with tubular differentiation. *Jpn. J. Cancer Res.*, **82**, 308–314.

KUNIYASU H, YASUI W, YOKOZAKI H, AKAGI M, AKAMA Y, KITAHARA K, FUJII K AND TAHARA E. (1994). Frequent loss of heterozygosity of the long arm of chromosome 7 is closely associated with progression of human gastric carcinomas. *Int. J. Cancer*, **59**, 597–600.

LEISTER I, WEITH A, BRÜDERLINE S, CZIEPLUCH C, KANGWANPONG D, SCHLAG P AND SCHWAB M. (1990). Human colorectal cancer: high frequency of deletions at chromosomes 1p35. *Cancer Res.*, **50**, 7232–7235.

LOUPART M-L, ARMOUR J, WALKER R, ADAMS S, BRAMMAR W AND VARLEY J. (1995). Allelic imbalance on chromosome 1 in human breast cancer. I. minisatellite and RFLP analysis. *Genes Chrom. Cancer*, **12**, 16–23.

MATHEW CGP, SMITH BA, THORPE K, WONG Z, ROYLE NJ, JEFFERYS AJ AND PONDER BAJ. (1987). Deletion of genes on chromosome 1 in endocrine neoplasia. *Nature*, **328**, 524–526.

MOLEY JF, BROTHER MB, FONG C-T, WHITE PS, BAYLIN SB, NELKIN B, WELLS SA AND BRODEUR GM. (1992). Consistent association of 1p LOH with pheochromocytomas from patients with multiple endocrine neoplasia type 2 syndromes. *Cancer Res.*, **52**, 770–774.

NAKAMURA Y, CARLSON M, KRAPCHO K AND WHITE R. (1988*a*). Isolation and mapping of a polymorphic DNA sequence (pMCT58) on chromosome 1p (D1S77). *Nucleic Acids Res.*, **16**, 9367.

NAKAMURA Y, CULVER M, SERGEANT L, LEPPERT M, O'CONNELL P, LATHROP GM, LALOUEL J-M AND WHITE R. (1988*b*). Isolation and mapping of a polymorphic DNA sequence (pYNZ2) on chromosome 1p (D1S77). *Nucleic Acids Res.*, **16**, 4747.

NAKATANI H, SAKAMOTO H, YOSHIDA T, YOKOTA J, TAHARA E, SUGIMURA T AND TERADA M. (1990). Isolation of an amplified DNA sequence in stomach cancer. *Jpn. J. Cancer Res.*, **81**, 707–710.

NAKATSURU S, YANAGISAWA A, ICHII S, TAHARA E, KATO Y, NAKAMURA Y AND HORII A. (1992). Somatic mutation of the *APC* gene in gastric cancer: frequent mutations in very well differentiated adenocarcinoma and signet-ring cell carcinoma. *Hum. Mol. Genet.*, **1**, 559–563.

PARKIN DM, LÄÄRÄ E AND MUIR CS. (1988). Estimates of the worldwide frequency of sixteen major cancers in 1980. *Int. J. Cancer*, **41**, 184–197.

SANO T, TSUJINO T, YOSHIDA K, NAKAYAMA H, HARUMA K, ITO H, NAKAMURA Y, KAJIYAMA G AND TAHARA E. (1991). Frequent loss of heterozygosity on chromosomes 1q, 5q, and 17p in human gastric carcinomas. *Cancer Res.*, **51**, 2926–2931.

SATO T, TANIGAMI A, YAMAKAWA K, AKIYAMA F, KASUMI F, SAKAMOTO G AND NAKAMURA Y. (1990). Allelotype of breast cancer: cumulative allele losses promote tumour progression in primary breast cancer. *Cancer Res.*, **50**, 7184–7189.

SCHLEIERMACHER G, PETER M, MICHON J, HUGOT J-P, VIELH P, ZUCKER J-M, MAGDELÉNAT H, THOMAS G AND DELATTRE O. (1994). Two distinct deleted regions in the short arm of chromosome 1 in neuroblastoma. *Genes Chrom. Cancer*, **10**, 275–281.

SIMON D, KNOWLES BB AND WEITH A. (1991). Abnormalities of chromosome 1 and loss of heterozygosity on 1p in primary hepatomas. *Oncogene*, **6**, 765–770.

STRICKLER JS, ZHENG J, SHU Q, BURGART LJ, ALBERTS SR AND SHIBATA D. (1994). p53 mutations and microsatellite instability in sporadic gastric cancer: When Guardians fail. *Cancer Res.*, **54**, 4750–4755.

TAKAHASHI E, HORI T, O'CONNELL P, LEPPERT M AND WHITE R. (1990). R-banding and nonisotopic *in situ* hybridization: precise localisation of the type II collagen gene (COL2A1). *Hum. Genet.*, **86**, 14–16.

TAKEDA O, HOMMA C, MASEKI N, SAKURAI M, KANDA N, SCHWAB M, NAKAMURA Y AND KANEKO Y. (1994). There may be two tumor suppressor genes on chromosome arm 1p closely associated with biologically distinct subtypes of neuroblastoma. *Genes Chrom. Cancer*, **10**, 30–39.

TAMURA G, KIHANA T, NOMURA K, TERADA M, SUGIMURA T AND HIROHASHI S. (1991). Detection of frequent p53 gene mutations in primary gastric cancer by cell sorting and polymerase chain reaction single-strand conformation polymorphism analysis. *Cancer Res.*, **51**, 3056–3058.

UCHINO S, TSUDA H, NOGUCHI M, YOKOTA J, TERADA M, SAITO T, KOBAYASHI M, SUGIMURA T AND HIROHASHI S. (1992). Frequent loss of heterozygosity at the *DCC* locus in gastric cancer. *Cancer Res.*, **52**, 3099–3102.

WEISSENBACH J, GYAPAY G, DIB C, VIGNAL A, MORISSETTE J, MILLASSEAU P, VAYSSEIX G AND LATHROP M. (1992). A second-generation linkage map of the human genome. *Nature*, **359**, 794–801.

WEITH A, MARTINSSON T, CZIEPLUCH C, BRÜDERLEIN S, AMLER LC AND BERTHOLD F. (1989). Neuroblastoma consensus deletion maps to 1p36.1–2. *Genes Chrom. Cancer.*, **1**, 159–166.

YANAGISAWA A, KATO Y, OHTAKE K, KITAGAWA T, OHASHI K, HORI M, TAKAGI K AND SUGANO H. (1991). c-Ki-*ras* point mutations in ductectatic-type mucinous cystic neoplasms of the pancreas. *Jpn. J. Cancer Res.*, **82**, 1057–1060.

YEH S-H, CHEN P-J, CHEN H-L, LAI M-Y, WANG C-C AND CHEN D-S. (1994). Frequent genetic alterations at the distal region of chromosome 1p in human hepatocellular carcinomas. *Cancer Res.*, **54**, 4188–4192.

YOKOTA J, YAMAMOTO T, MIYAJIMA N, TOYOSHIMA K, NOMURA N, SAKAMOTO H, YOSHIDA T, TERADA M AND SUGIMURA T. (1988). Genetic alterations of the c-*erb*B-2 oncogene occur frequently in tubular adenocarcinoma of the stomach and are often accompanied by amplification of the v-*erb*A homologue. *Oncogene*, **2**, 283–287.